# Editorial: Sex differences in sleep and circadian rhythms

**DOI:** 10.3389/fnins.2025.1583842

**Published:** 2025-03-25

**Authors:** Maria M. Hadjimarkou, Jessica A. Mong

**Affiliations:** ^1^School of Psychology, University of Sussex, Brighton, United Kingdom; ^2^Department of Pharmacology, University of Maryland School of Medicine, Baltimore, MD, United States

**Keywords:** estrous cycle, gonadal hormones, excessive daytime sleepiness, sleep deprivation, four core genotype

Although research on sleep and circadian rhythms has been expanding, there remains a gap in our understanding of sex differences in this area. This Research Topic explores existing studies on sex differences in sleep and circadian rhythms.

Covassin et al. evaluated the risk of cardiovascular disease, cancer, and all-cause mortality in men and women with Obstructive Sleep Apnoea (OSA), using excessive daytime sleepiness (ESS) as a predictor. In men, ESS was associated with a reduced risk of hypertension, a finding not observed in women. Both men and women with ESS had a greater risk of developing diabetes. However, after a median follow-up of 6.2 years (range: 4.5–8.1 years), an increased risk of all-cause mortality was found in women with OSA and ESS but not in men. This suggests that while OSA primarily affects men, the long-term impact of ESS is more severe in women. These findings highlight the importance of ESS as a symptom and the need for further research to understand the underlying mechanisms.

Tir et al. discuss the implications of neglecting key variables in sleep and circadian rhythm research. A random sample of 1,355 studies published between 1979 and 2019 was reviewed for the reporting of sample size, age, sex, gender, ethnicity, education level, socioeconomic status (SES), and profession. Although there has been an upward trend in reporting and analyzing demographic variables in sleep and chronobiology research, the majority (90%) of studies reported only age or sex, while fewer than 25% included data on other demographic factors. While controlling for these factors is relatively straightforward in animal studies, human research would benefit from collecting data on both biological sex and gender, along with other demographic variables, to better understand the interplay of biological, psychological, social, and environmental influences on sleep and circadian rhythms in an increasingly diverse population.

Swift et al. discuss the persistent biases in sleep research, particularly the assumption that physiological processes in men and women are comparable or unaffected by the estrous cycle. Studies that have examined sex differences in sleep indicate that in naturally cycling female rats, NREM and REM sleep are reduced during proestrus and estrus, with a corresponding increase in wakefulness, contributing to observed sex differences. Ovariectomy (OVX) increases NREM and/or REM sleep, while the reintroduction of gonadal hormones restores the pre-OVX sleep profile. These sex differences appear to be driven by the organizational effects of gonadal hormones early in life (Cusmano et al., 2014).

Possible mechanisms influencing these differences include the suppression of activity in preoptic nuclei neurons and the suppression of prostaglandin D2, both of which lead to increased wakefulness. Estradiol can act on arousal centers to promote wakefulness, impacting both sides of the sleep-wake flip-flop switch. Additionally, progesterone, through its metabolites and its effects on the GABAA receptor or heat-sensitive neurons in the preoptic area, may also play a role in sleep regulation.

Further research on the actions of estradiol, progesterone, and other hormones such as prolactin may provide deeper insights into the mechanisms involved in sleep regulation during the estrous cycle. Future studies should also explore sex differences in elements of sleep architecture, such as sharp-wave ripples and sleep spindles, which are critical for learning and memory. Additionally, research on the role of hormones in the development of conditions such as PTSD—studied predominantly in males but not in females—remains an important area of investigation.

Ralston et al.'s review further emphasizes the urgency of understanding sex differences in sleep and circadian rhythms, particularly in relation to sleep and wake-related conditions, as well as the disparities in male and female animal models of neurological conditions such as Huntington's (Chiem et al., 2024), Alzheimer's (Campbell et al., 2024), and Autism Spectrum Disorder (Lord et al., 2022).

In healthy mice, females exhibit more consolidated sleep but sleep less overall than males. However, this sex difference disappears following gonadectomy. The four-core genotype mouse model provides a framework for disentangling the effects of gonadal hormones and sex chromosomes. In this model, the Sry gene is removed from the sex chromosomes and expressed in an autosome, resulting in XY and XX males that carry the Sry gene and have testes, and XY and XX females that lack the Sry gene and have ovaries. Importantly, sex-linked genes influence recovery from sleep loss, with male mice recovering more quickly from sleep deprivation than female mice (Paul et al., 2006).

This is particularly intriguing given findings by Shi et al. (2024), who reported sex differences in the cortical transcriptome of 11 genes following 6 h of sleep deprivation. Specifically, male and female mice exhibited reduced expression of genes such as BDNF, Fosb, and Fosl2, which are essential for neuronal function, learning, memory, and overall plasticity. Previous studies have also identified greater susceptibility to sleep deprivation in female rodents, as evidenced by poorer performance in discriminative avoidance tasks (Fernandes-Santos et al., 2012) and spatial learning tasks (Hajali et al., 2012) compared to males. These findings appear to extend to humans as well - Santhi et al. (2016) reported that women are more sensitive to circadian rhythms and sleep deprivation, exhibiting greater impairments in nighttime performance compared to men. This suggests that women may experience greater adverse effects from sleep deprivation than men, with potential implications for lifestyle choices.

Overall, this Research Topic underscores the need for further studies to enhance our understanding of sex differences in sleep and circadian rhythms. While gonadal hormones and the estrous cycle contribute to these differences, there are also hormonal effects beyond the estrous cycle that impact sleep and arousal centers. Furthermore, evidence suggests that sleep deprivation affects men and women differently, with greater susceptibility observed in women. Future research should continue to explore these differences to develop more tailored interventions for sleep disorders across sexes.
